# What is the role of Intramarrow penetration in managing intrabony defects—A Systematic Review

**DOI:** 10.1007/s44445-026-00130-6

**Published:** 2026-03-12

**Authors:** Aaron Nedungadi, Sharath Shetty, Anita Kulloli, Santosh Martande, Pooja V, Dileep Sharma

**Affiliations:** 1 Department of Periodontology, Dr D Y Patil Dental College & Hospital, Dr D Y Patil Vidyapeeth, Pimpri, Pune, India; 2 Discipline of Oral Health, School of Health Sciences, College of Health, Medicine and wellbeing, The Universty of Newcastle, Ourimbah, Australia

**Keywords:** Intrabony defects, Intramarrow penetration, Decortication, Periodontitis, Periodontal regeneration

## Abstract

Intramarrow Penetration (IMP) has recently gained attention for its potential to enhance periodontal healing with regenerative approaches for osseous deformities such as intrabony defects. This systematic review was conducted to evaluate the effect of IMP in enhancing periodontal regeneration of intrabony defects when used in conjunction with or without the use of biomaterials. A comprehensive search was performed across databases including PubMed, Scopus, Cochrane, Lilacs, and Google Scholar. Studies were selected based on the inclusion of clinical outcomes such as probing pocket depth (PPD), clinical attachment level (CAL), and bone fill following the application of IMP with Open Flap debridement (OFD) with or without biomaterials. Studies were assessed for risk of bias using the Cochrane RoB 2 tool. The quality of evidence was assessed using the GRADE approach. Initial search yielded 194 studies which eventually resulting in five eligible studies for inclusion in the review. Four of the five included studies reported that IMP significantly improved clinical and radiographic outcomes, including reduced PPD, increased CAL, and enhanced bone fill. IMP can be a potential adjunct to OFD for periodontal regeneration in intrabony defects, offering advantages such as improved healing and enhanced bone regeneration. However, as the quality of evidence is very low a definitive clinical recommendation cannot be made. Further well-designed multicentre studies are necessary to validate these findings.

## Introduction

Periodontitis is a chronic inflammatory disease characterized by clinical attachment loss and the formation of osseous deformities, such as furcation and intrabony defects (Reynolds et al. 2015). Specifically, intrabony defects also known as vertical defects pose a significant risk for disease progression and continued attachment loss, if left untreated (Pham 2021). The primary goals of periodontal treatment include reducing pocket depth, controlling risk factors, and converting intrabony defects into maintainable sites to prevent further deterioration. Treatment protocols typically begin with mechanical debridement i. e., scaling and root debridement (SRD) to reduce bacterial load, sometimes supplemented with local or systemic pharmacologic therapies. Subsequent modalities may include resective or regenerative procedures, with latter being the preferred approach, as it aims to restore the structure and function of periodontium (alveolar bone, periodontal ligament, and cementum) (Bowers et al. 1982). Deep intrabony defects present a significant clinical challenge as they need surgical intervention to create an optimal environment for new attachment formation and periodontal regeneration (Saudi HIE-MA and El Ghaysh 2017).

Open Flap Debridement (OFD), is a surgical approach that leads to reparative healing characterized by the formation of a long junctional epithelium (Crea et al. 2014; Nickles et al. 2009). To enhance regenerative outcomes in periodontal defects, various biomaterials and techniques have been introduced. Bone graft materials including autografts, allografts, xenografts, and alloplasts are commonly used to provide a scaffold for clot stabilization and bone remodelling (Dumitrescu 2011; Saleh et al. 2014). Specific bone replacement grafts (BRGs) currently used clinically includes demineralized freeze-dried bone allograft (DFDBA), GTR with barrier membranes, and biologics like Enamel Matrix Derivatives(EMD) and recombinant human platelet-derived growth factor-BB (rhPDGF-BB) combined with β-tricalcium phosphate (β-TCP) (Kao et al. 2015). Platelet concentrates, including leukocyte-rich PRF (L-PRF) and titanium-prepared PRF (T-PRF), are also utilized due to their prolonged release of growth factors enhancing their regenerative potential (Ravi and Santhanakrishnan 2020). To further improve regenerative outcomes, Intramarrow Penetration (IMP), also known as decortication, is often incorporated into regenerative periodontal procedures.

Intramarrow Penetration (IMP) technique involves creating perforations in the cortical bone to expose the underlying cancellous bone, thereby promoting localized bleeding (Lundgren et al. 2000). This facilitates clot formation whilst also promoting release of cytokines and growth factors, attracting progenitor cells, osteoblasts, and new blood vessels within the defect site. These biological events are known to enhance revascularization and bone regeneration. Additionally, the perforations serve as conduits for blood vessels and cells, ensuring rapid access to the treated area and accelerating the healing process (Sharma et al. 2023; Greenstein et al. 2009).

Previous studies suggest that IMP can improve the outcomes of regenerative procedures including enhanced predictability of OFD in treating intrabony defects. However, the clinical benefits of IMP remain uncertain, as no clinical trials have specifically investigated its adjunctive use with OFD in managing intrabony defects (Crea et al. 2008; Pagliaro et al. 2008). The incorporation of adjunctive techniques like IMP, along with biomaterials and biologics represents a step forward in improving predictable and successful regenerative outcomes. Thus, this systematic review aims to determine role of intramarrow penetration in surgical management of intrabony defects.

## Materials and methods

This systematic review was conducted following the PRISMA guidelines (Preferred Reporting Items for Systematic Reviews and Meta‑analyses) (Page et al. 2020). The protocol was registered on PROSPERO [Registration number: CRD420250653540 accessible on https://www.crd.york.ac.uk/PROSPERO/view/CRD420250653540]. The review question was “Are there variations in the effectiveness of Open Flap Debridement (OFD) combined with Intramarrow Penetration (IMP), with or without biomaterials, compared to Open Flap Debridement (OFD) alone in promoting periodontal regeneration of intrabony defects?”.

The PICOS framework for this research question can be summarised as—P (Population): adult human patients diagnosed with periodontitis and present with intrabony periodontal defects requiring surgical intervention; I (Intervention): Open Flap Debridement (OFD) combined with Intramarrow Penetration (IMP), administered either with biomaterials or without biomaterials; C (Comparison): Open Flap Debridement (OFD) without IMP; O (Outcomes): Clinical attachment level (CAL) gain, probing pocket depth (PPD) reduction, bone gain or defect fill, and linear bone growth; S (Study Design): Randomized controlled trials (RCTs).

### Search strategy

A thorough and systematic literature search was conducted using relevant keywords/Medical Subject Headings (MeSH) and their combinations with Boolean operators (OR, AND) across five online biomedical databases: PubMed, Scopus, Cochrane, Lilacs, and Google Scholar with the last search completed in September 2025. The search string(s) used to identify studies from these databases are listed in Table 1.

**Table 1:** Search strategy for the literature search

Database	Search String
PubMed	#1 ("Intramarrow"[All Fields] AND ("penetrability"[All Fields] OR "penetrable"[All Fields] OR "penetrate"[All Fields] OR "penetrated"[All Fields] OR "penetrates"[All Fields] OR "penetrating"[All Fields] OR "penetration"[All Fields] OR "penetrations"[All Fields]) AND ("intrabony"[All Fields] AND ("abnormalities"[MeSH Subheading] OR "abnormalities"[All Fields] OR "defects"[All Fields] OR "defect"[All Fields] OR "defect s"[All Fields] OR "defected"[All Fields] OR "defective"[All Fields] OR "defectively"[All Fields] OR "defectives"[All Fields]))) AND ((humans[Filter]) AND (english[Filter]))
	# 2 ("intramarrow"[All Fields] AND ("penetrability"[All Fields] OR "penetrable"[All Fields] OR "penetrate"[All Fields] OR "penetrated"[All Fields] OR "penetrates"[All Fields] OR "penetrating"[All Fields] OR "penetration"[All Fields] OR "penetrations"[All Fields])) AND ((humans[Filter]) AND (english[Filter]))
Scopus	(ALL (decortication) AND ALL (intrabony defects) AND ALL (intramarrow penetration))
Cochrane library	(intra marrow penetration) AND (intrabony defects)
LILACS	intramarrow penetration AND db:("LILACS") AND instance:"lilacsplus"
Google scholar	("intramarrow penetration") AND ("intrabony defects")

### Inclusion and exclusion criteria

Studies were considered for inclusion if they were published in English, randomised controlled trials (RCTs) in humans with intrabony defect(s) treated using specifically Open flap debridement with intramarrow penetration (with or without biomaterials). All non-English language publications, study designs apart from RCTs, and other bone defect types and/or flap designs led to exclusion from this review. Additionally, the primary outcomes eligible for inclusion were probing pocket depth, clinical attachment loss (CAL), bone fill and bone density. Studies that failed to report on at least some of these outcome measures were also excluded from this review.

### Data extraction

The relevant data from the included studies were extracted and recorded in a pre-piloted data extraction spreadsheet. This included authors, country, study design, sample size, patient demographics (age/gender), control/intervention groups, follow-up intervals, and the effectiveness of intramarrow penetration for treating intrabony defects (Table 2). The data extraction process was conducted independently by two review authors (AN and SM), and any discrepancies were resolved through discussions with other authors (DS and AK) to achieve consensus.

**Table 2:** Summary of findings from included studies involving IMP for Intrabony defects

Author/Country	Study design	Study population			Follow-up (months)	Outcome measures	Radiographic protocol utilised	Conclusion
		Total sample (N) Groups (n)	Age (Yrs)	M:F ratio				
Crea et al. 2014 ; USA	RCT	N = 41 T-OFD + IMP (n = 28); C- OFD (n = 13)	52.3 ± 6.9 (mean)	20:21	12	The IMP group showed: – Higher clinical bone gain (3.07–1.74 mm) vs control group (1.76–2.71 mm) – CAL gain ≥ 2 mm in 93% of sites vs control 62% – Significant reduction of Radiographic defect Depth in 2-wall defects	Periapical radiographs used to calculate radiographic defect depth—Distance from most coronal point of the residual bone crest to the bottom of the defect	IMP significantly enhanced both clinical and radiographic outcomes, especially in mandibular defects
Saudi al. 2017 ; Egypt	RCT	N = 30 T- OFD + IMP (n = 10); T- OFD + NcHA granules (n = 10) C- OFD (n = 10);	≥ 28 years	NR	3 & 6	The IMP group showed (at 6 months) statistically significant improvement in – CAL (3.6 ± 1.57) vs. control (1.6 ± 1.71 mm) – Bone density (13.6 ± 2.79) vs. control (5.4 ± 2.63) No Statistically significant difference between IMP and NcHA granules use compared to control	Digital panoramic radiographs used to measure mean grey level with custom-designed Visual Basic program	IMP significantly enhanced outcomes in treating intrabony defects in chronic periodontitis patients
Saini et al. 2020; India	RCT	N = 32 T -OFD + IMP + DFDBA (n = 16); C- OFD + DFDBA (n = 16)	36.25 ± 9.44 (mean)	16:16	3, 6 & 9	The IMP group showed statistically significant improvement (9 mths) in – PPD (3.72 ± 0.87 mm) vs. control (3.00 ± 0.72 mm) – CAL (3.67 ± 0.86 mm) vs. control (2.99 ± 0.74 mm) – Radiographic defect area 0.77 vs. 2.13 ± 0.50) – Bone fill (%) (39.47 ± 13.92% vs. 19.29 ± 14.24%) – Linear bone growth (1.41 ± 0.54 mm vs. 0.62 ± 0.49 mm)	Intraoral periapical radiographs with customised stent to calculate measurements related to defect area % (1mmx1mm radiographic grids) and distance from most coronal point of the residual bone crest to the bottom of the defect	Addition of decortication to the DFDBA graft significantly enhanced healing potential and clinical outcomes across all measured timepoints (0–3, 6 & 9 months)
Bharti et al. 2021; India	Split mouth RCT	N = 20 T- OFD + IMP + PRF (n = 10); C- OFD + PRF (n = 10)	30–55 years	NR	3 & 6	The IMP group showed statistically significant improvement (at 6 mths) – PPD (4.10 + 0.45 mm) vs. control (2.90 + 0.51 mm) – CAL (4.05 ± 0.43 mm) vs. control (2.90 ± 0.61 mm) – Linear Bone fill (1.17 ± 0.20 vs. 0.58 ± 0.07 mm)	Radiovisiography (RVG) images used to calculate measurements—distance from most coronal point of the residual bone crest to the bottom of the defect	Combination of IMP and PRP enhanced the clinical outcomes in periodontal intrabony defects compared to PRP alone
Sharma et al. 2023 ; India	RCT	N = 20 T- OFD + IMP + A-PRF (n = 10); C- OFD + A-PRF (n = 10)	22–60 years	NR	6	The IMP group showed no statistically significant improvement (at 6 mths) – – GI (1.4 ± 0.52 vs. 1.4 ± 0.52) – PI (1.4 ± 0.52 vs. 1.5 ± 0.53) – PPD (6.4 ± 1.65 vs. 5.9 ± 1.73 mm) – CAL (5.9 ± 1.45 vs. 5.4 ± 1.58 mm) – No significant gain in radiographic bone fill or height	Intraoral periapical radiographs with customised stent to calculate measurements related to defect area % (1mmx1mm radiographic grids) and distance from most coronal point of the residual bone crest to the bottom of the defect	Although both groups showed significantly improved clinical outcomes compared to baseline, no statistically significant improvements when compared to A-PRF alone (inter-group)

*PPD* Pocket probing depth; *CAL* Clinical attachment loss; *DFDBA* Demineralized Freeze-Dried Bone Allograft; *GI* Gingival index; *PI* Plaque index; *NcHA* Nanocrystalline hydroxyapatite; *RCT* Randomised controlled Trial; *IMP* Intramarrow penetration; *OFD* Open flap debridement; *A-PRF* Advanced platelet-rich fibrin; *PRF* platelet-rich fibrin; *NR* Not reported; *T* Test group; *C* Control group

### Risk of bias assessment

Each study was assessed for risk of bias independently using the Cochrane Collaboration’s Risk of Bias (RoB 2) tool, with reviewers (AN and PV) performing the evaluations (Sterne et al. 2019). Any inconsistencies in bias assessment were resolved through discussions with a third reviewer (DS).

### Qualitative synthesis

**Fig. 1:**
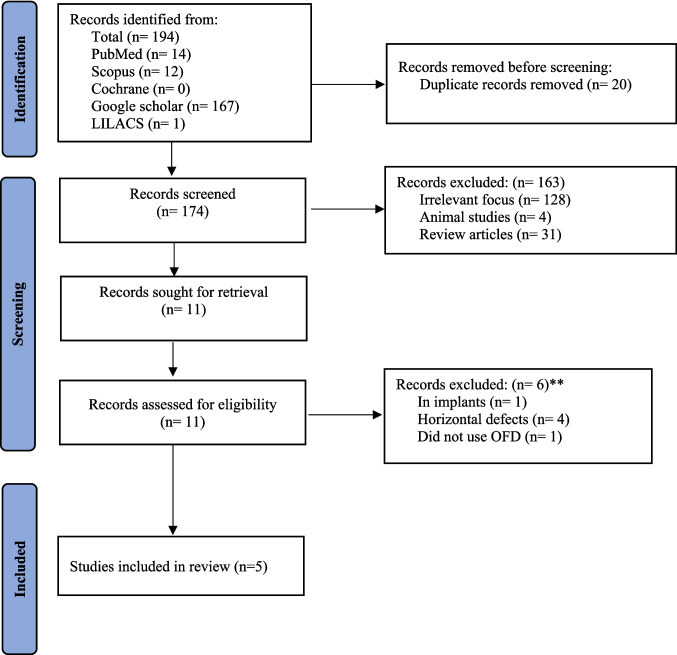
PRISMA Flow chart illustrating the literature search and screening of studies

**Fig. 2:**
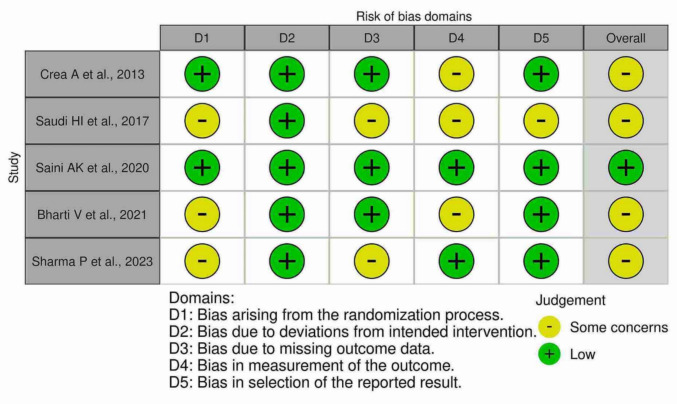
Risk of bias assessment of included studies

**Fig. 3:**
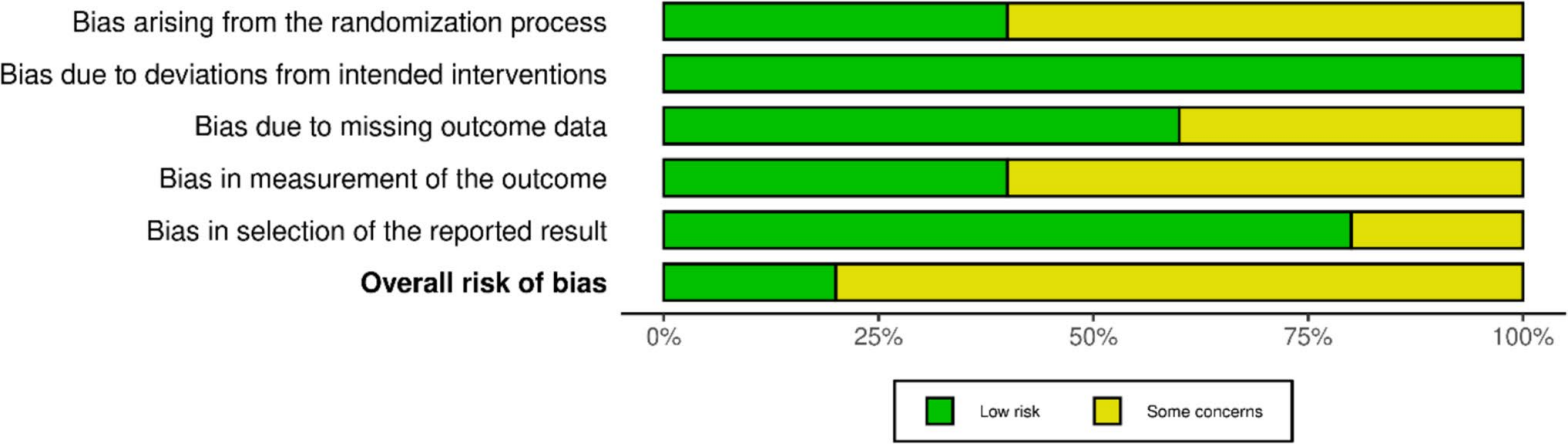
Overall risk of bias of included studies

The qualitative analysis of the data from the eligible studies was performed by summarizing the results and presenting them in tabular form. A flowchart was used to illustrate the process of the literature search (Fig. 1). The critical appraisal of the methodological quality (risk of bias) of the included studies is presented in Figs. 2 and 3.

The overall quality of evidence for the outcome (effectiveness of the IMP for intrabony defects) from the included RCTs was assessed using the GRADE approach by two independent review authors (SS and DS). The GRADE analysis was conducted in accordance with the criteria specified by Murad et al., (Murad et al. 2017) which is specifically designed for SRs without meta-analysis. The details of the GRADE assessment for the RCT studies are presented in Table 3.

**Table 3:** Certainty of evidence for the included studies

Outcomes assessed	Study design	Initial grading	Studies	Reasons for downgrading the evidence	Quality of evidence
Effectiveness of Intramarrow penetration for intrabony defects	RCT	High	Crea A et al Saudi HI et al Saini AK et al Bharti V et al Sharma P et al	Downgrading factors: (−3) RoB: (−1) Serious Indirectness: (−1) Serious Imprecision: (−1) Serious Inconsistency: Not Serious Publication bias: Not detected	Very Low ⊕ ⊖ ⊖ ⊖

## Results

A comprehensive literature search initially yielded 194 records that reduced to 174 articles after removal of duplicates. Subsequent titles and abstract screening led to 163 irrelevant records being excluded. The titles and abstracts screening were performed independently by two authors (AN and SS), with a high level of agreement, as indicated by Cohen’s Kappa value (κ = 0.92). In cases of discrepancies or disagreements, a third review author (DS) was consulted to reach a consensus. Subsequent full text evaluation of 11 articles led to inclusion of 5 studies that met the established eligibility criteria (Fig. 1).

### Characteristics of the included studies

The included studies were published between 2013 and 2023 with the majority of them conducted in India (3/5) (Sharma et al. 2023; Saini et al. 2020; Bharti  et al. 2021), and one each in the USA (Crea et al. 2014), and Egypt (Saudi HIE-MA and El Ghaysh 2017). All the studies (n = 5) employed a randomized controlled trial (RCT) design as per the review protocol. The sample sizes varied between 20 and 41 participants, age ranging between 22 to 60 years.

The intervention in all the studies involved OFD with IMP (n = 2), (Saudi HIE-MA and El Ghaysh 2017; Crea et al. 2014) OFD and IMP with PRF (n = 2), (Sharma et al. 2023; Bharti et al. 2021) and Demineralized Freeze-Dried Bone Allograft (DFDBA) (n = 1) (Saini et al. 2020). The control group utilised in the studies were not uniform and typically consisted of either OFD alone or OFD with nanocrystalline hydroxyapatite, DFDBA, or PRF. Key clinical outcomes were reported, including probing pocket depth (PPD), clinical attachment level (CAL), plaque index (PI), gingival index (GI), and bone fill. Follow-up periods varied across the studies, with outcomes being evaluated from 3 months up to 12 months.

IMP demonstrated significant improvements in key clinical parameters, including PPD, CAL, and radiographic bone fill particularly in mandibular defects (Crea et al. 2014; Saini et al. 2020). However, another study reported that the addition of IMP showed better clinical outcomes but the radiographic improvements were not always significantly superior when compared to OFD with other adjuncts such as A-PRF (Sharma et al. 2023). This finding suggests that while IMP enhances the clinical parameters, its radiographic effectiveness may be influenced by the biomaterials used in combination, necessitating further investigation into optimal combinations for improved patient outcomes. The secondary outcomes, as reported by Crea et al. and Saini et al. did not exhibit statistically significant differences, indicating that the effects of IMP were analogous to those observed with OFD alone (Crea et al. 2014; Saini et al. 2020).

### Methodological quality of the included studies

The methodological assessment determined low risk of bias for one study and some concerns for four studies mainly associated with randomization, measurement of outcome and missing outcome data (Figs. 2 and 3).

### Certainty/Quality of evidence

The baseline GRADE analysis was initially considered high, as only randomized controlled trials (RCTs) (n = 5) were included in this systematic review. However, due to the presence of three downgrading factors—namely "Risk of Bias" (RoB), "indirectness," and "imprecision", the overall quality of the evidence was rated as "very low" for the outcome of this review. The downgrading factors (RoB, indirectness, inconsistency, imprecision, and risk of publication bias) were all considered when assessing the overall quality of the evidence (Table 3).

## Discussion

This systematic review evaluated the role of IMP incorporation with OFD for the treatment of intrabony defects with or without the combination of biomaterials. The findings indicate that IMP is a valuable adjunct to OFD, enhancing both clinical and radiographic outcomes when used with Platelet-Rich Fibrin (PRF) and Demineralized Freeze-Dried Bone Allograft (DFDBA). Furthermore, IMP can improve periodontal regeneration whilst reducing PPD, increasing CAL gain, and enhancing bone fill.

Combining IMP with a full-thickness flap on intact alveolar bone did not cause any pathological changes in bone quality or quantity once homeostasis was restored. In treating intrabony defects, IMP may also promote clot formation and maturation, crucial for periodontal regeneration (Crea et al. 2014; Baloul et al. 2011). Two included studies suggested that IMP provided more predictable and stable results than OFD alone, particularly for cases with complex defects although the results were not statistically significant (Sharma et al. 2023; Bharti et al. 2021). Saini et al. found the radiographic bone density to be similar with or without the addition of IMP (Saini et al. 2020). Furthermore, it was reported that the addition of IMP with OFD did not show significant differences when compared to OFD with nanocrystalline hydroxyapatite granules in terms of bone density and mobility (Saudi HIE-MA and El Ghaysh 2017). This indicates that while IMP is a beneficial addition, the choice of including a biomaterial might influence the level and density of bone fill. Additionally, it may be speculated that there may be no additional benefits of IMP when it is used with advanced regenerative materials, as opposed to its use with OFD alone. Although all studies included in the review employed IMPs, yet none analysed its effect as an independent variable. However, no adverse events were attributed to IMPs, reinforcing their procedural safety.

A recent meta-analysis that included four studies systematically evaluated the of cortical perforation in management of intrabony defects clinically. Across four randomized controlled trials included, probing depth (PD) reduction did not differ significantly between perforation and control groups (Pesce et al. 2025). Similarly, clinical attachment level (CAL) and recession (REC) outcomes showed no meaningful differences. However, radiographic parameters—such as defect depth and CEJ-to-defect distance—demonstrated modest improvements in the perforation group, suggesting localized benefits without definitive clinical superiority. Our systematic review did include two additional studies whilst excluding one due to it being an unpublished thesis available in Italian language.

Clinical improvements following regenerative therapy are often supported by radiographic evidence of defect fill, typically with at least 50% or greater improvement (Reynolds et al. 2003; Darby and Morris 2013). Bone fill was noted to be significantly higher when using IMP, within the included studies (Crea et al. 2014). This may be attributed to IMP and its role in facilitating blood circulation and release of growth factors, promoting localised revascularization and bone regeneration (Greenstein et al. 2009). Additionally, IMP can facilitate bone regeneration by enhancing osteoblast migration to the defect site (Verdugo et al. 2012). Perforation of the cortical bone is also reported to improve the interface between the recipient site and graft material, resulting in enhanced clinical and radiographic outcomes (Dayangac et al. 2016). This process is activated by local release of osteoprogenitor cells and osteoinductive factors through Frost’s Regional Accelerated Phenomenon (RAP), thereby improving the quality of the newly formed bone (Babrawala et al. 2021). Findings from animal studies have also suggested that IMP promotes localized enhancement of cancellous bone turnover and periodontal ligament activity, likely due to RAP (Frost 1983). While the molecular mechanisms behind these effects remain unclear, animal study indicated that increased RANKL expression plays a key role in the process and IMP alone can enhance bone formation during the later stages of alveolar bone healing (Baloul et al. 2011). Furthermore, a recent rodent study reported that IMP increases the expression of nerve growth factor that promotes osteogenesis around bone graft through regulation of JNK/c-Jun pathway thereby contributing to bone regeneration (Jiao et al. 2025).

Quantification of bone gain or bone fill using radiographic images are commonly utilised across the literature relating to regenerative therapy as they are non-invasive. In the included studies, three used the intraoral periapical radiography (Crea et al. 2014; Sharma et al. 2023; Saini et al. 2020) while one study used radiovisiography to quantify bone gain (Bharti et al. 2021). Both these approaches used parallelling or long cone technique that is considered to produce least distortion and often preferred when reproducibility and quantitative measures are needed (Yen and Yeung 2023). However, one included study (Saudi HIE-MA and El Ghaysh 2017) used panoramic radiography that is known to possess inherent distortion (magnification) and rarely utilised in regenerative surgeries (Yeo et al. 2002) Consequently, the possible influence of radiographic protocols reported in included papers on the overall outcomes cannot be understated.

IMP is typically performed by creating or drilling several small, round perforations on the surface of cortical bone related to the osseous defect using rotary instrument. One of the primary advantages of IMP is its cost-effectiveness and minimal surgical complexity making it a practical approach for clinical incorporation. While the risks of potential complications that relate to bone surgery such as postoperative pain, swelling, osteonecrosis due to overheating of bone and tooth (root) injury does exist, none of these have been peviously reported in relation to IMP.

This systemic review is the first to compare the role of IMP in combination with most common flap technique i.e., open flap debridement. However, the review has some limitations. Included studies showed significant heterogeneity in terms of small sample sizes, varying follow-up periods (3–12 months), methodological approaches and outcome measures. For example, the control group in only two of the included studies were OFD alone while three studies utilised a biomaterial along with IMP as control that may have positively influenced the reported outcomes. This heterogeneity in study design makes it difficult to ascertain the therapeutic effect of IMP alone. Additionally, the radiographic protocols and quantification of ‘bone fill’, varied across the studies with a range of parameters measured including bone density, linear bone fill and linear bone growth. This is also evident in the risk of bias assessment (RoB-2, Figs. 2 and 3) which confirmed that some concerns existed in four of five included studies, particularly regarding the randomization process and measurement of outcomes affecting the positive clinical effects reported. Overall, this variability along with patient demographics and surgical technique(s) utilised across the studies limits the direct comparison of results or consideration of conducting meta-analyses.

Future research in this domain should involve multicentre randomised controlled trials with standardized methodologies and outcome measures to improve the robustness and generalizability of the findings relating to IMP use. Additionally, consistent follow-up and more comprehensive data collection will assist in an objective comparison and build evidence for the long-term benefits of IMP in the regenerative periodontal therapy, specifically for intrabony defect management.

## Conclusion

IMP is a promising adjunct to traditional OFD in periodontal regenerative procedures for managing intrabony defects. Incorporation of IMP may offer potential advantages such as enhanced bone regeneration, reduced surgical trauma, and faster recovery with or without the use of biomaterials. As noted in our review, IMP could improve clinical outcomes and bone fill with optimal patient selection and technique. However, the certainty of evidence remains very low, highlighting the need for further research to warrant clinical application with or without other biomaterials.

## Data Availability

No datasets were generated or analysed during the current study.

## References

[CR1] Babrawala I, Chickanna R, Prabhuji MLV, Mampuzha S, Khanna D, Sali D. Treatment of intrabony defects by decortication with CeraboneTM and DFDBA - A randomised controlled trial. J Dental Health Oral Res 02(02)

[CR2] Baloul SS, Gerstenfeld LC, Morgan EF, Carvalho RS, Van Dyke TE, Kantarci A (2011) Mechanism of action and morphologic changes in the alveolar bone in response to selective alveolar decortication–facilitated tooth movement. Am J Orthod Dentofacial Orthop 139(4):S83–S101 21435543 10.1016/j.ajodo.2010.09.026

[CR3] Bowers GM, Schallhorn RG, Mellonig JT (1982) Histologic evaluation of new attachment in human intrabony defects: a literature review. J Periodontol 53(8):509–514 6750076 10.1902/jop.1982.53.8.509

[CR4] Crea A, Dassatti L, Hoffmann O, Zafiropoulos GG, Deli G (2008) Treatment of intrabony defects using guided tissue regeneration or enamel matrix derivative: a 3‐year prospective randomized clinical study. J Periodontol 79(12):2281–2289 19053918 10.1902/jop.2008.080135

[CR5] Crea A, Deli G, Littarru C, Lajolo C, Orgeas GV, Tatakis DN (2014) Intrabony defects, open‐flap debridement, and decortication: a randomized clinical trial. J Periodontol 85(1):34–42 23537123 10.1902/jop.2013.120753

[CR6] Darby IB, Morris KH (2013) A systematic review of the use of growth factors in human periodontal regeneration. J Periodontol 84(4):465–476 22612370 10.1902/jop.2012.120145

[CR7] Dayangac E, Araz K, Oguz Y, Bacanli D, Caylak B, Uckan S (2016) Radiological and histological evaluation of the effects of cortical perforations on bone healing in mandibular onlay graft procedures. Clin Implant Dent Relat Res 18(1):82–88 24889104 10.1111/cid.12238

[CR8] Dumitrescu A (2011) Bone grafts and bone graft substitutes in periodontal therapy. Chem Surg Period Ther 73–144

[CR9] Frost HM (1983) The regional acceleratory phenomenon: a review. Henry Ford Hosp Med J 31(1):3–9 6345475

[CR10] Greenstein G, Greenstein B, Cavallaro J, Tarnow D (2009) The role of bone decortication in enhancing the results of guided bone regeneration: a literature review. J Periodontol 80(2):175–189 19186957 10.1902/jop.2009.080309

[CR11] Jiao Y, Liu Y, Li X, Han N, Liu S, Du J et al (2025) Cortical perforation promotes bone regeneration by enhancing nerve growth factor secretion. Biochem Biophys Res Commun 755:151562 40043613 10.1016/j.bbrc.2025.151562

[CR12] Kao RT, Nares S, Reynolds MA (2015) Periodontal regeneration – intrabony defects: a systematic review from the AAP regeneration workshop. J Periodontol. 10.1902/jop.2015.130685 25216204 10.1902/jop.2015.130685

[CR13] Lundgren AK, Lundgren D, Hammerle CH, Nyman S, Sennerby L (2000) Influence of decortication of the donor bone on guided bone augmentation. An experimental study in the rabbit skull bone. Clin Oral Implants Res 11(2):99–106 11168200

[CR14] Murad MH, Mustafa RA, Schünemann HJ, Sultan S, Santesso N (2017) Rating the certainty in evidence in the absence of a single estimate of effect. Evid Based Med 22(3):85–87 28320705 10.1136/ebmed-2017-110668 PMC5502230

[CR15] Nickles K, Ratka‐Krüger P, Neukranz E, Raetzke P, Eickholz P (2009) Open flap debridement and guided tissue regeneration after 10 years in infrabony defects. J Clin Periodontol 36(11):976–983 19807821 10.1111/j.1600-051X.2009.01474.x

[CR16] Page MJ, Moher D, Bossuyt PM, Boutron I, Hoffmann TC, Mulrow CD et al. (2021) PRISMA 2020 explanation and elaboration: updated guidance and exemplars for reporting systematic reviews. BMJ n160 10.1136/bmj.n160 PMC8005925 33781993

[CR17] Pagliaro U, Nieri M, Rotundo R, Cairo F, Carnevale G, Esposito M et al (2008) Clinical guidelines of the Italian society of periodontology for the reconstructive surgical treatment of angular bony defects in periodontal patients. J Periodontol 79(12):2219–2232 19053910 10.1902/jop.2008.080266

[CR18] Pesce P, Canullo L, Testori T, Mastroianni A, Fabbro MD, Menini M (2025) The clinical effect of bone perforations in periodontal regeneration and alveolar socket preservation: a systematic review with meta-analysis. Clin Oral Investig. 10.1007/s00784-025-06152-4 39814954 10.1007/s00784-025-06152-4 PMC11735581

[CR19] Pham TAV (2021) Intrabony defect treatment with platelet-rich fibrin, guided tissue regeneration and open-flap debridement: a randomized controlled trial. J Evid Based Dent Pract 21(3):101545 34479673 10.1016/j.jebdp.2021.101545

[CR20] Ravi S, Santhanakrishnan M (2020) Mechanical, chemical, structural analysis and comparative release of PDGF-AA from L-PRF, A-PRF and T-PRF - an in vitro study. Biomater Res 24(1) 10.1186/s40824-020-00193-4 PMC7488539 32944280

[CR21] Reynolds MA, Aichelmann‐Reidy ME, Branch‐Mays GL, Gunsolley JC (2003) The efficacy of bone replacement grafts in the treatment of periodontal osseous defects. A systematic review. Ann Periodontol 8(1):227–265 14971256 10.1902/annals.2003.8.1.227

[CR22] Reynolds MA, Kao RT, Nares S, Camargo PM, Caton JG, Clem DS et al (2015) Periodontal regeneration — intrabony defects: practical applications from the AAP regeneration workshop. Clin Adv Periodontics 5(1):21–29 32689725 10.1902/cap.2015.140062

[CR23] Saini AK, Tewari S, Narula SC, Sharma RK, Tanwar N, Sangwan A (2020) Comparative clinical and radiographic evaluation of demineralized freeze-dried bone allograft with and without decortication in the treatment of periodontal intrabony defects: a randomized controlled clinical study. Quintessence Int 51(10):822–837 32661522 10.3290/j.qi.a44921

[CR24] Saleh RG, Allah OMG, Tokhey HME, El-Guindy HM (2014) Evaluation of hydroxyapatite nanoparticles with and without silver nanoparticles in the treatment of induced periodontitis in dogs. J Am Sci 12(10):21–33

[CR25] Saudi SA, El Ghaysh AM (2017) The validity of intramarrow penetration with open flap debridement in the treatment of intrabony defects in patients with chronic peridontitis. EC Dental Science 13(6):266–276

[CR26] Sharma P, Manjunath SRG, Gummaluri SS, Kunche L (2023) Intramarrow penetration synergized with advanced platelet-rich fibrin in periodontal regeneration: a randomized controlled trial. J Indian Soc Periodontol 27(3):301–307 37346857 10.4103/jisp.jisp_199_22 PMC10281316

[CR27] Sterne JAC, Savović J, Page MJ, Elbers RG, Blencowe NS, Boutron I, et al. (2019) RoB 2: a revised tool for assessing risk of bias in randomised trials. BMJ l4898 10.1136/bmj.l4898 31462531

[CR28] Bharti V, Nagi PK, Singh M (2021) Comparative evaluation of Platelet Rich Fibrin with and without Intra-Marrow Penetration in the treatment of Intra-Bony defects – a clinical and radiographic study. Int J Res Health Allied Sci 7(2):87–94. https://ijrhas.com/uploadfiles/20vol7issue2pp87-%2094.20210424083006.pdf

[CR29] Verdugo F, D’Addona A, Ponton J (2012) Clinical, tomographic, and histological assessment of periosteal guided bone regeneration with cortical perforations in advanced human critical size defects. Clin Implant Dent Relat Res 14(1):112–120 20491815 10.1111/j.1708-8208.2009.00235.x

[CR30] Yen M, Yeung AWK (2023) The performance of paralleling technique and bisecting angle technique for taking periapical radiographs: a systematic review. Dent J 11(7):155 10.3390/dj11070155 PMC10378420 37504221

[CR31] Yeo DK, Freer TJ, Brockhurst PJ (2002) Distortions in panoramic radiographs. Aust Orthod J 18(2):92–98 12462686

